# ANN and LEFM-Based Fatigue Reliability Analysis and Truck Weight Limits of Steel Bridges after Crack Detection

**DOI:** 10.3390/s22041580

**Published:** 2022-02-17

**Authors:** Lei Nie, Wei Wang, Lu Deng, Wei He

**Affiliations:** 1 College of Civil Engineering, Hunan University, Changsha 410082, China; nielei@hnu.edu.cn (L.N.); wang_wei@hnu.edu.cn (W.W.); wei_he@hnu.edu.cn (W.H.); 2 Key Laboratory for Damage Diagnosis of Engineering Structures of Hunan Province, Hunan University, Changsha 410082, China

**Keywords:** steel girder bridge, truck weight limit, fatigue reliability analysis, Monte Carlo simulation, linear elastic fracture mechanics, artificial neural network

## Abstract

Fatigue of steel bridges is a major concern for bridge engineers. Previous fatigue-based weight-limiting method of steel bridges is founded on the Palmgren–Miner’s rule and S-N curves, which overlook the effect of existing cracks on the fatigue life of in-service steel bridges. In the present study, based on the theory of linear elastic fracture mechanics, a framework combining the artificial neural networks and Monte Carlo simulations is proposed to analyze the fatigue reliability of steel bridges with the effects of cracks and truck weight limits considered. Using the framework, a new method of setting the gross vehicle weight limit for in-service steel bridges with cracks is proposed. The influences of four key parameters, including the average daily truck traffic, the gross vehicle weight limit, the violation rate, and the detected crack size, on the fatigue reliability of a steel bridge are analyzed quantitatively with the new framework. Results show that the suggested framework can enhance the fatigue reliability assessment process in terms of accuracy and efficiency. The method of setting gross vehicle weight limits can effectively control the fatigue failure probability to be within 2.3% according to the desired remaining service time and the detected crack size.

## 1. Introduction

Fatigue of steel bridges has always been one of the major concerns of bridge engineers. An increasing number of steel bridges are suffering from fatigue cracks under the action of cyclic heavy trucks, which may threaten the safety of bridges. Therefore, it is urgent and significant to research the fatigue problem of steel bridges under overloaded truck traffic and take reasonable measures for vehicle weight limits.

The traditional idea for vehicle weight limits is checking and comparing the value of the stress induced by the overloaded truck with that induced by the design load [1]. Many countries or regions have issued corresponding regulations to check whether a truck is permitted to cross a bridge. For example, the Bridge Formula B is widely applied in the United States to judge whether a heavy vehicle can cross a bridge [2]. The Bridge Formula B is given as follows:

(1) $$W=2224\left(\frac{0.305BN}{N-1}+12N+36\right)$$

where *W* denotes the permissible weight in newtons of any set of multi-axles; *N* represents the number of axles in the selected axle set; and *B* denotes the length between the two outermost axles in the selected axle set. However, some researchers criticized that the bridge damage caused by repeated truck loadings is not taken into account when the Bridge Formula B is adopted for permitting check [3,4]. Deng and Yan [4] proposed a new weight-limiting method that can consider the effect of fatigue damage under the cyclic loadings of trucks based on the theory of the S-N curve and Palmgren–Miner’s rule. Furthermore, Wang et al. [5] established a random traffic load model to consider the simultaneous existence of multiple trucks, and proposed a new method for determining the truck weight limit according to fatigue reliability analyses. However, these studies were performed on the basis of the theory of the S-N curve and Palmgren–Miner’s rule, in which the effect of original cracks on the fatigue life of steel bridges can hardly be taken into account [6,7].

The linear elastic fracture mechanics (LEFM) is an important branch of fracture mechanics, and its feasibility and reliability in fatigue analysis of steel bridges with cracks have been fully verified [8]. The location and the size of structural cracks can be considered when the theory of LEFM is adopted for analyzing the remaining fatigue life of steel bridges [6], which contributes to a more accurate fatigue analysis of in-service steel bridges with cracks. Due to the strong uncertainty and randomness during the process of crack growth, many scholars conducted probabilistic analyses on the fatigue problem of steel bridges based on the LEFM. Pipinato et al. [9] used the probabilistic methods and LEFM to calculate the fatigue reliability of a highway steel girder bridge under the dual effects of earthquake loads and traffic loads. Guo and Chen [10] investigated the growth of fatigue cracks of an old steel box girder bridge, in which fatigue reliability analyses on the basis of the LEFM were performed according to the long-term stress monitoring data. Leander and Al-Emrani [11] proposed a probabilistic method for the fatigue evaluation of steel bridges on the basis of the LEFM, and investigated the impact of different modeling options on the reliability index of details. Although a large number of studies have been conducted to analyze crack growth in steel bridges based on the LEFM, few studies have been conducted to investigate the effects of traffic load conditions on the fatigue reliability of steel bridges with cracks based on the theory of LEFM, especially for the case of implementing truck weight limits. Therefore, it is necessary to provide an efficient method that can quickly analyze the fatigue reliability of steel bridges with cracks under different traffic load conditions, and provide valuable suggestions for setting reasonable truck load limits accordingly.

In recent years, many new artificial intelligence-based methods have been developed and applied to bridge health monitoring and fatigue analysis [12,13,14,15]. Guo et al. [12] used Kohonen neural network and long short-term memory neural network to evaluate the bridge health state. Huynh [13] proposed a new autonomous vision-based bolt-looseness detection method for splice bolted connections based on the Faster RCNN. Lu et al. [14] proposed a machine learning-based framework to calculate the fatigue reliability of steel bridge decks. Yan et al. [15] proposed a probabilistic machine learning method for fatigue analysis of steel bridges under the action of overloaded trucks.

In the present study, a new framework is proposed for fatigue reliability analysis of steel bridges with cracks, which can be used to quickly calculate the fatigue failure probability of steel bridges under truck weight limits according to different traffic load parameters. A typical 30.4-m-span steel-concrete composite girder bridge with a crack at the bottom flange of the girder was utilized as an instance to demonstrate the proposed framework. According to the transportation survey data from Wisconsin of the United States, a numerical model of random traffic load under load-limiting conditions is first established. Then, the limit state function of fatigue failure is obtained using the theory of LEFM when taking into account the crack detection error and the uncertainty process of crack growth. Finally, the ANN-MCS method is introduced to analyze fatigue reliability of the crack under consideration. Effects of four key parameters, including the average daily truck traffic, the gross vehicle weight limit, the violation rate, and the crack size, on the time-varying fatigue reliability of the steel bridge are quantitatively analyzed. Based on the analysis, a new method was proposed to set the gross vehicle weight limit for in-service steel bridges with cracks.

## 2. Bridge Model and Fatigue Detail

### 2.1. Bridge Model under Consideration

**Figure 1:**
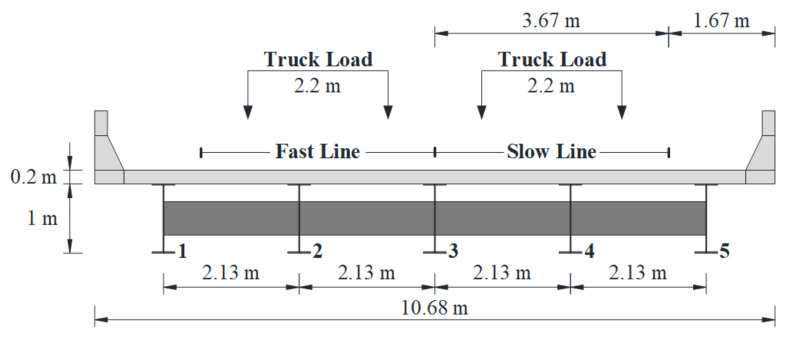
Cross-section of the bridge.

**Figure 2:**
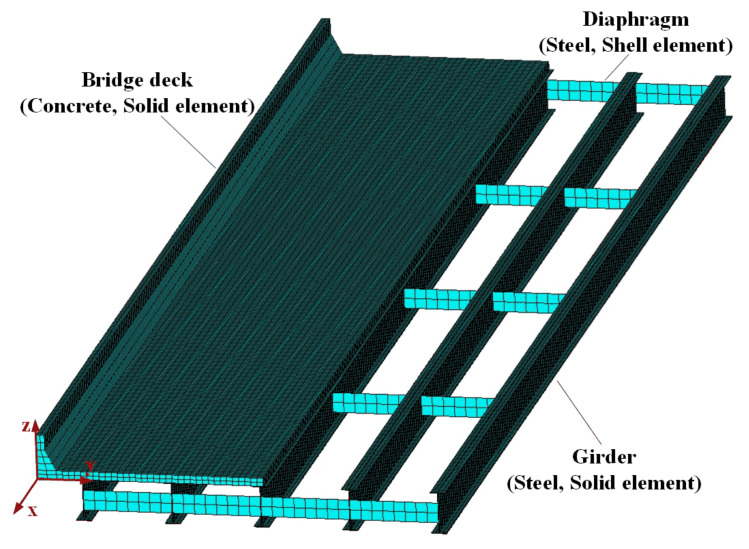
3-D FE bridge model.

In the present study, a simply supported girder bridge with five steel girders was utilized as the bridge model, which was designed on the basis of the AASHTO LRFD specification [16]. The bridge span length is 30.4 m (100 ft), and the width is 10.68 m (35 ft). Five diaphragms, including three intermediate diaphragms and two end diaphragms, are evenly distributed along the bridge to bond the adjacent girders. The typical cross-section of the bridge and the corresponding main dimensions are demonstrated in Figure 1. A three-dimension (3-D) finite element (FE) model of the adopted bridge, as illustrated in Figure 2, was constructed using ANSYS. Solid elements were utilized for the bridge deck slab, the guardrails, and the five girders, and shell elements were utilized for the diaphragms in the FE model. There are a total of 64,768 elements in the FE model. The nodes at one end of the bottom of the girders are constrained in the *x*, *y*, and *z* directions and the nodes at the other end of the bottom of the girders are constrained in the y and z directions as shown in Figure 2. It should be noted that the FE model adopted in the present study was also used for fatigue analysis in previous studies [15,17]. Two loading cases were considered in the study, in which the truck with a wheel gauge of 2.2 m was assumed to travel along the bridge in the middle of each traffic lane, as shown Figure 1.

### 2.2. Fatigue Detail of Interest

**Figure 3:**
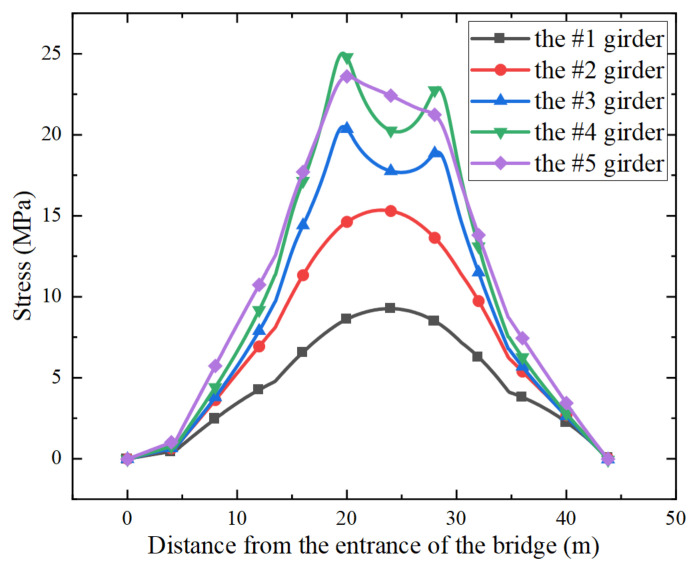
Stress histories at the weld between the bottom flange and web at the mid-span of the 1st girder to the 5th girder.

**Figure 4:**
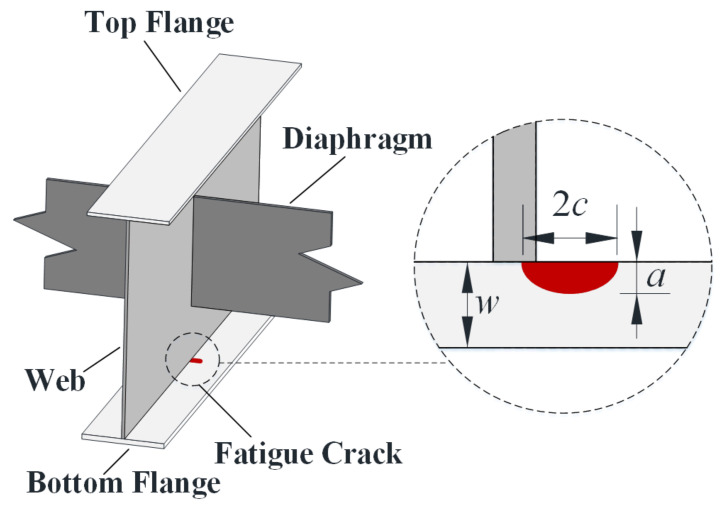
Fatigue crack position at the mid-span of the 4th girder.

**Figure 5:**
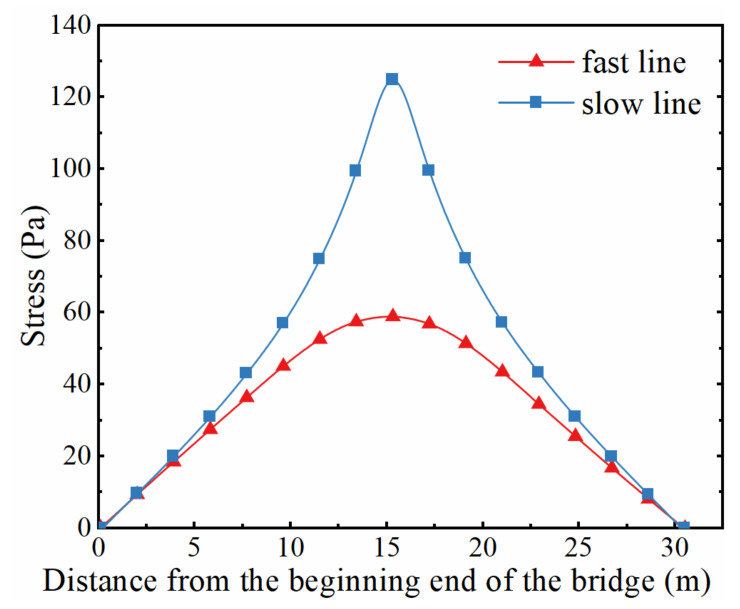
Longitudinal stress influence lines of the fatigue detail.

Through numerical simulations, it was found that when the fatigue truck in AASHTO *LRFD* 2017 (HS20-44) was applied along the slow lane, the weld between the bottom flange and web at the mid-span of the 4th girder in Figure 1 experienced the maximum stress range, as shown in Figure 3. Therefore, the 4th girder was adopted for the fatigue reliability analysis in the study. In fact, previous studies indicated that the bending stresses of fatigue details at the mid-span govern the fatigue design of small and medium span bridges [15,18]. It should be noted that the fatigue detail of interest in the present study, which corresponds to the detail category B in the AASHTO LRFD [16], was also adopted as a typical fatigue detail in other studies [5,15]. The location and the dimensions of the crack in the fatigue detail are illustrated in Figure 4, in which *w* represents the thickness of the bottom flange, adopting a value of 22.2 mm, and the crack shape is presumed to be semi-elliptical, with a depth of *a* and a width of 2*c*. The stress influence lines of the fatigue detail under consideration were obtained when a pair of loads (0.5 N per wheel) were applied along the slow line and along the fast line, respectively, as illustrated in Figure 5.

## 3. Traffic Load Model and Stress History of the Fatigue Detail

### 3.1. Traffic Load Data

**Figure 6:**
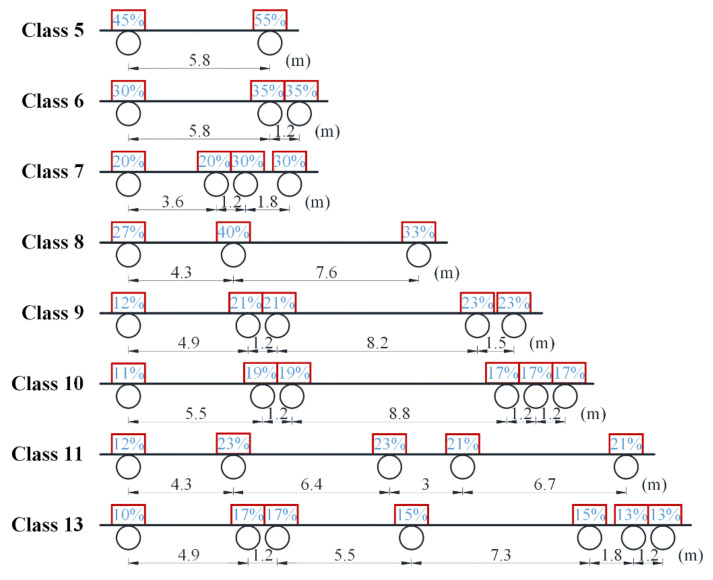
The axle weight ratio and the mean axle spacing of truck classes.

The random traffic load model used in this paper was generated according to the traffic load data recorded by a WIM (weigh-in motion) station in Wisconsin of the United State performed by Haider and Harichandran [19]. They investigated the traffic load data and observed that the gross vehicle weight (GVW) of trucks specified in the FHWA vehicle classifications can be well fitted with a combination of two normal distributions. The statistical parameters of the traffic load in Wisconsin, i.e., the proportion and the statistical distribution parameters of the GVW of each truck class, provided by Haider and Harichandran, are summarized in Table 1, in which *P* denotes the percentage of the truck class in the traffic flow; *μ_i_*, *σ_i_*, and *p_i_* (*i* = 1, 2) denote, respectively, the average value, the standard deviation, and the percentage of corresponding distribution. It is worth noting that as the statistical parameters provided by Haider and Harichandran do not contain the data of truck Class 4 and Class 12, the portions of trucks represented by Class 4 and Class 12 were incorporated into Class 5 and Class 13 in Table 1, respectively. This merging operation could lead to a little conservative result of the vehicle-induced fatigue damage because truck Class 4 and Class 12 account for a small percentage (7.48% in total) of the traffic flow and the GVWs of truck Class 4 and Class 12 are generally smaller than the GVWs of truck Class 5 and Class 13, respectively. A similar merging operation was also adopted in the research conducted by Wang et al. [5]. The axle weight ratio, namely, the percentage of the axle weight to the GVW, and the mean axle spacing of each truck class are summarized in Figure 6, in which the circles represent the axles of each truck class, and the number marked above the circle represents the corresponding axle weight ratio.

**Figure 7:**
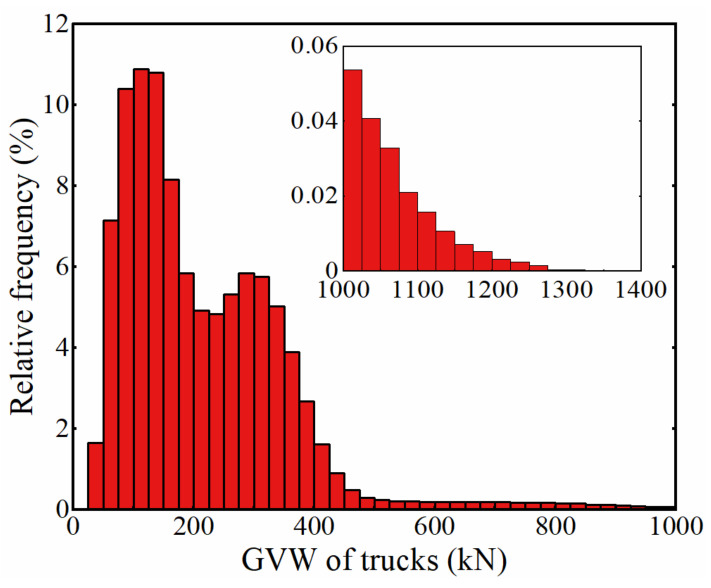
Distribution of GVWs of trucks in the simulated traffic flow.

As the fatigue damage caused by trucks with a GVW below 30 kN is negligible [14], these trucks were excluded from the simulated traffic flow in this paper. The distribution of the GVWs of trucks in the simulated traffic flow is shown in Figure 7, from which it can be seen that the trucks with a GVW exceeding 1200 kN accounted for less than 0.01% of the simulated traffic. As the frequency of these trucks was extremely low in the simulated traffic, it was inconvenient and unnecessary to analyze the corresponding information about these trucks [20]. Therefore, trucks with a GVW exceeding 1200 kN were also excluded from the simulated traffic in the subsequent analysis. Actually, the GVWs of trucks in the simulated truck traffic ranges from 30 kN to 1200 kN in the following analysis.

### 3.2. Stress History of the Fatigue Detail under Truck Weight Limits

The stress time histories of the fatigue detail under the cyclic loadings of trucks introduced in the previous section were obtained through numerical simulations. As indicated in the traffic survey by Nowak [21], it is rare for a truck to follow another one in the same lane with a distance of less than 30 m. Therefore, the values of the headway distance, which is defined as the distance from the rear axle of one truck to the front axle of the truck behind, were set as random numbers larger than the span length (30.4 m) in the present study.

The effects of five key parameters, including the *t*, ADTT, PFL, GVWL, and VR, on the generated random traffic loads were considered. The *t* represents the expected remaining service time of the bridge in years. The ADTT refers to the average number of daily truck traffic before the implementation of the truck weight limit. The PFL represents the proportion of trucks in the fast lane and the GVWL refers to the value of the gross vehicle weight limit for the bridge. The VR refers to the violation rate and a value of 0.2 for VR means that 20% of all trucks that exceed the GVWL would cross the bridge illegally.

**Figure 8:**
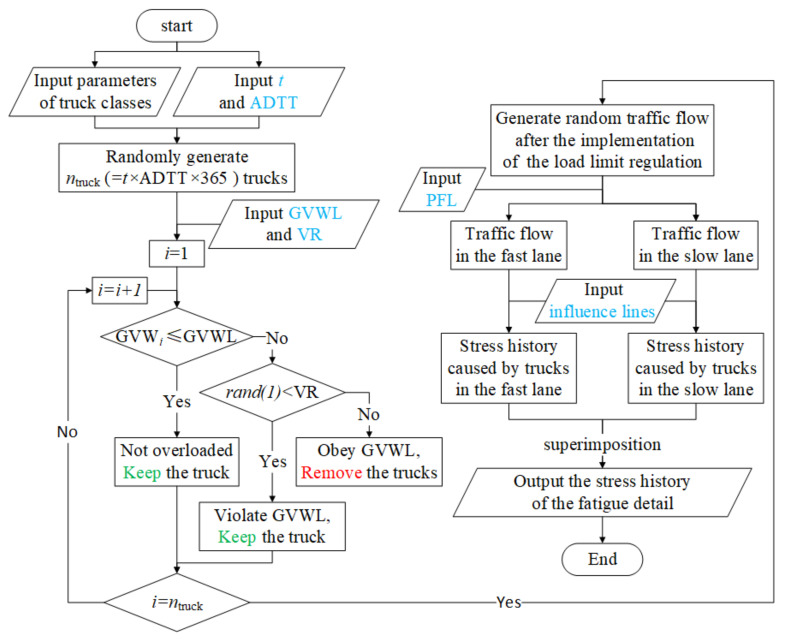
Flowchart of generating the stress history of the fatigue detail.

A four-step procedure was carried out to generate the stress history of the fatigue detail under the random traffic loads. Firstly, according to the parameters of trucks provided in Table 1 and Figure 6, *n*_truck_ (*n*_truck_ = *t* × ADTT × 365) trucks were randomly generated as the traffic loads before implementing the weight limit. Secondly, overloaded trucks that comply with the weight limit were removed from the traffic flow generated in the first step, in which random numbers, uniformly distributed between 0 and 1, were utilized to judge whether a truck would cross the bridge. If the random number was greater than the VR, then it was assumed that the truck driver complied with the weight limit regulation and gave up crossing the bridge. Thirdly, according to the value of PFL, the traffic flow was divided into two parts, namely, the traffic flow in the fast lane and that in the slow lane. The stress histories caused by trucks in the two lanes were computed separately with the two influence lines shown in Figure 5. Finally, the stress histories caused by trucks in the two lanes were superimposed to produce the stress history of the fatigue detail. It should be noted that the starting points of the two signals are assumed coincident during the superimposition operation. A value of 0.15 was adopted as the impact factor to consider the dynamic effects of trucks when generating the stress history of the fatigue detail. The flowchart for calculating the stress history of the fatigue detail under consideration is exhibited in Figure 8. It should be noted that the function *rand(1)* in the flowchart is used to generate a random number uniformly distributed from 0 to 1.

## 4. Method of LEFM

The theory of LEFM was demonstrated to perform well for fatigue analysis of steel bridges with cracks [8]. The Paris Law, which is one of the most representative rule based on the theory of LEFM, describes the relationship between the rate of growth of a crack and stress-intensity factor (*K*) induced by the cyclic loads, as show in Equation (2).

(2) $$\frac{da}{dN}=C{\left(\Delta K\right)}^{m}$$

where *a* denotes the crack size; *N* denotes the number of cycles; *C* and *m* are constants related to material properties, and *m* = 3 for carbon steels; *K* denotes the stress-intensity factor, which describes the magnitude of the stress and strain values near the crack tip; and Δ*K* is the stress-intensity factor range. The crack size of the fatigue detail under consideration can be calculated by solving the differential equation.

The stress intensity factor *K* is influenced by many factors, including the crack shape, the crack size, and the component size. According to the research by Newman and Raju [22], the *K* for a semi-elliptical surface crack under the action of the remote tensile stress can be calculated to be:

(3) $$K=F_{s}\left(\frac{a}{w},\frac{a}{c},\frac{c}{b},\phi \right)\sqrt{\frac{1}{Q}}\sigma \sqrt{\pi a}$$

where *σ* denotes the remote uniform-tension stress. A reasonable estimation of *Q* can be obtained in Equation (4) when *a*/*c* ≤ 1.

(4) $$Q=1+1.464{\left(\frac{a}{c}\right)}^{1.65}$$

The stress-intensity boundary-correction factor in Equation (3) for surface cracks was taken as

(5) $$F_{s}=\left[M_{1}+M_{2}{\left(\frac{a}{w}\right)}^{2}+M_{3}{\left(\frac{a}{w}\right)}^{4}\right]g_{1}f_{\phi }f_{W}$$

where

(6) $$M_{1}=1.13-0.09\left(\frac{a}{c}\right)$$

(7) $$M_{2}=-0.54+\frac{0.89}{0.2+a/c}$$

(8) $$M_{3}=0.5-\frac{1}{0.65+a/c}+14\cdot {\left(1-\frac{a}{c}\right)}^{24}$$

(9) $$g_{1}=1+\left[0.1+0.35{\left(\frac{a}{w}\right)}^{2}\right]{\left(1-\sin\phi \right)}^{2}$$

(10) $$f_{\phi }={\left[{\left(\frac{a}{c}\right)}^{2}\cos^{2}\phi +\sin^{2}\phi \right]}^{1/4}$$

(11) $$f_{W}={\left[\sec\left(\frac{\pi c}{2b}\sqrt{\frac{a}{w}}\right)\right]}^{1/2}$$

In these equations, *a* and *c* represent the depth and the half-length of the surface crack; *ϕ* represents the parametric angle of the ellipse, adopting *π*/2 for the point at the maximum depth; and *w* and *b* represent the thickness and the half-width of the bottom flange, taking a value of 22.2 mm and 177.8 mm, respectively. The aspect ratio of the crack (*a*/*c*) was assumed to be 0.62 in the present study [11]. It is worth noting that, as the purpose of the study mainly focuses on investigating the growth of the crack along the thickness direction of the bottom plate, the crack size in the present study denotes the crack depth of *a* shown in Figure 4.

A set of stress cycles were extracted from the stress history caused by traffic loads using the rainflow-counting algorithm. As it is complicated to use a set of stress ranges with variable amplitudes to calculate the crack growth rate, the stress ranges were equivalently converted into a constant stress range (*S*_re_) to simplify the calculation process [7,8]. The equivalent stress range (*S*_re_) is computed as:

(12) $$S_{\mathrm{re}}={\left(\sum f_{i}S_{r,i}^{3}\right)}^{\frac{1}{3}}$$

where $f_{i}$ is the frequency of the *i*th stress range *S*_r,*i*_. Therefore, according to Equation (3), the stress-intensity factor range was calculated as:

(13) $$\Delta K=F_{s}\sqrt{\frac{1}{Q}}S_{\mathrm{re}}\sqrt{\pi a}.$$

## 5. ANN-MCS Method for Fatigue Reliability Analysis

### 5.1. Limit State Function

The expression of Δ*K* in Equation (13) was utilized in the Paris Law to facilitate integration. Therefore, the crack growth rate was calculated as:

(14) $$\frac{da}{dN}=C{\left(F_{s}\sqrt{\frac{1}{Q}}S_{\mathrm{re}}\sqrt{\pi a}\right)}^{m}.$$

Integration was performed with respect to the crack size growing from *a*_1_ to *a*_2_, and Equation (15) was obtained [7].

(15) $$\int_{a_{1}}^{a_{2}}\frac{1}{{\left(F_{s}\sqrt{\frac{1}{Q}}\sqrt{\pi a}\right)}^{m}}da=CS_{\mathrm{re}}^{m}\left(N_{2}-N_{1}\right)$$

In Equation (15), *a*_1_ and *a*_2_ denote the size of the crack after being subjected to a number of *N*_1_ and *N*_2_ stress cycles, respectively. The left side of Equation (15) represents the fatigue damage accumulation corresponding to the process of the crack size increasing from *a*_1_ to *a*_2_, as shown in Equation (16).

(16) $$\Psi \left(a_{2},a_{1}\right)=\int_{a_{1}}^{a_{2}}\frac{1}{{\left(F_{s}\sqrt{\frac{1}{Q}}\sqrt{\pi a}\right)}^{m}}da$$

Therefore, the limit state function (LSF) of fatigue failure of the detail is [7]:

(17) $$\mathrm{LSF}=\Psi \left(a_{c},a_{0}\right)-CS_{\mathrm{re}}^{m}\left(N_{t}-N_{0}\right)$$

in which *a*_0_ denotes the actual size of the crack at the moment of crack measurement; *a*_c_ denotes the critical crack size; *N*_0_ is the number of stress cycles corresponding to *a*_0_; and *N_t_* is the number of stress cycles after the crack inspection for *t* years. As the time (*t*) mentioned in this paper started from the detection time of the crack, *N*_0_ equals zero in the present study [7]. Accordingly, the failure probability (*P*_f_) and the corresponding reliability index (*β*) was calculated as:

(18) $$P_{f}=P\left(\mathrm{LSF}\leq 0\right)\;\approx \Phi (-\beta ).$$

### 5.2. Uncertainties of Parameters

According to the study in Zhao et al. [23], the actual crack size (*a*_0_) of fatigue details can be described by a lognormal distribution with the detected crack size (*a*_dtc_) as the mean value and 0.25 as the coefficient of variation (CoV). In this study, the parameter *C* in the Paris law was demonstrated to follow a lognormal distribution with $3{.92\;\times \;10}^{-12}$ (MPa^3^$\sqrt{m}$)^−1^ as the mean value and 0.62 as the CoV, and the parameter *m* was considered to be a constant (*m* = 3) [7,8,24]. Additionally, the key traffic-related parameters, including the ADTT, GVWL, and VR, were treated as constants and investigated in the parametric-analysis sections. The statistical characteristics of parameters in the limit state function are summarized in Table 2. It is worth noting that the critical crack size in the present study was taken as the thickness of the bottom flange (*w*) [7,8].

### 5.3. Procedure of the ANN-MCS Method

The computer used in the present study was an ordinary personal computer (PC) that had an Intel^®^ Core^™^ i5-9600K processor with 3.70 GHz base frequency and a random-access memory (RAM) with 16 GB capacity. Through trial calculations, it was found that the reliability analysis was time-consuming, especially in the process of generating random traffic loads and running the numerical integration in the Monte Carlo simulation (MCS).

**Figure 9:**
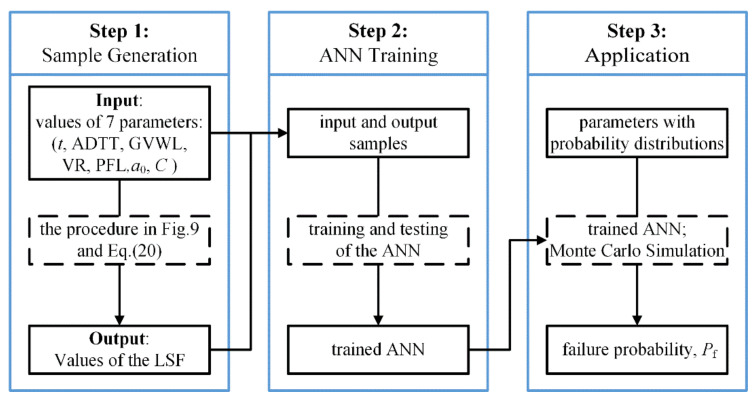
Procedure of the ANN-MCS method for reliability analysis.

The artificial neural network (ANN) was proven to have excellent performance in fitting highly nonlinear and complex processes, and was utilized to enhance computation efficiency of calculating the LSF. The procedure for the LSF calculation based on the artificial neural network and MCS (namely, the ANN-MCS method) is shown in Figure 9, from which it can be seen that the procedure consists of three steps, including the sample-generation step, the ANN-training step, and the application step.

## 6. Construction of the ANN

### 6.1. Generating Training-Set Samples

The procedure of generating training-set samples for the ANN training is introduced in this section, in which each sample consists of seven input parameters (*t*, ADTT, GVWL, PFL, VR, *a*_0_, and *C*) and one output parameter (LSF). For the convenience of description, these seven input parameters were divided into traffic-characteristic parameters (ADTT, GVWL, PFL, and VR) and non-traffic-characteristic parameters (*t*, *a*_0_, and *C*). The ranges of input parameters are summarized in Table 3. It should be noted that the procedure of generating random traffic loads, which is determined by traffic-characteristic parameters, is the most time-consuming part. To make a balance between the accuracy and computation efficiency, the training-set samples were generated by a four-step procedure. Firstly, multiple sets of traffic-characteristic parameters were generated. Many experimental design methods, including the completely random design method, orthogonal design method, and Latin square design method, can be used as the reference to select traffic-characteristic parameters to improve efficiency [25]. In general, more sets of traffic-characteristic parameters help generate a higher-precision neural network. In the present study, 351 sets of pseudo-random traffic-characteristic parameters for the training-set inputs were generated through the completely random design and the orthogonal design method. Secondly, according to the process shown in Figure 8, the corresponding 351 sets of 50-year-period random traffic loads were generated, and the stress histories of the fatigue detail under the action of the generated random traffic loads were calculated. Thirdly, for each set of traffic-characteristic parameters, 500 sets of non-traffic-characteristic parameters were generated separately and randomly, and a total of 175,500 (351 × 500 = 175,500) sets of input parameters were obtained. Finally, for each set of input parameters, the value of LSF was calculated as the output parameter. It is worth noting that the stress history of the fatigue detail generated in the second step was utilized in this step. Additionally, if *t* was less than 50 years, the stress history was intercepted correspondingly.

### 6.2. ANN Training and Testing

**Figure 10:**
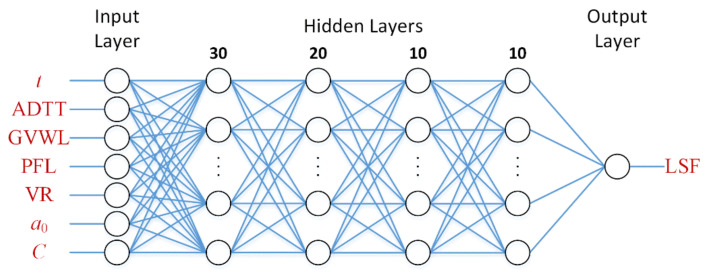
Architecture of the ANN.

The architecture of the neural network adopted in this paper is shown in Figure 10, from which it can be seen that the network is composed of an input layer, four hidden layers, and an output layer. The number of neurons of the first to the fourth hidden layers was set to 30, 20, 10, and 10, respectively, and the sigmoid function was chosen as the activation function of hidden layer neurons.

The mean square error (MSE) was specified as the loss function to assess the performance of the neural network. The maximum number of epochs for training the neural network was set to 1000 and the training process completed while the maximum number of epochs reached or the loss function (MSE) of the neural network was minimized to zero.

A Bayesian regularization method, which combines the Levenberg–Marquardt algorithm and a Bayesian framework proposed by MacKay, was applied to train the neural network in the present study [26,27]. The advantage of this method is that the optimal regularization parameters were determined in an automated manner to improve the generalization ability and avoid over-fitting of the network.

**Figure 11:**
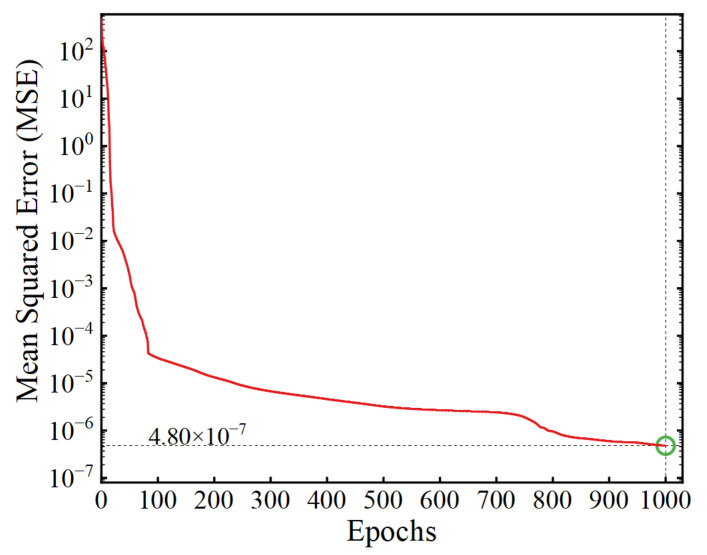
The variation of loss function with the increasing of epochs.

**Figure 12:**
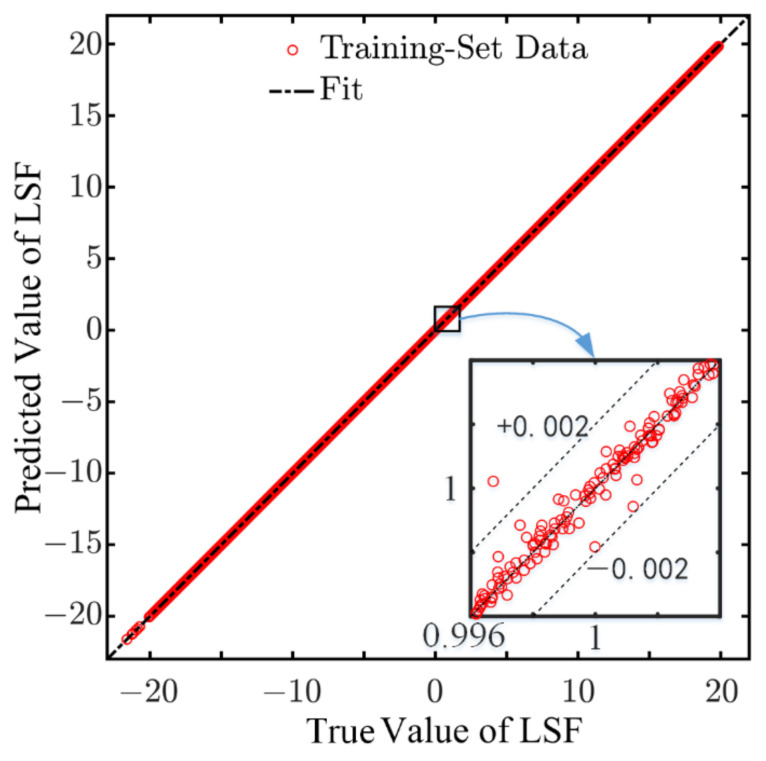
Comparison of the predicted value of the ANN and the true value.

**Figure 13:**
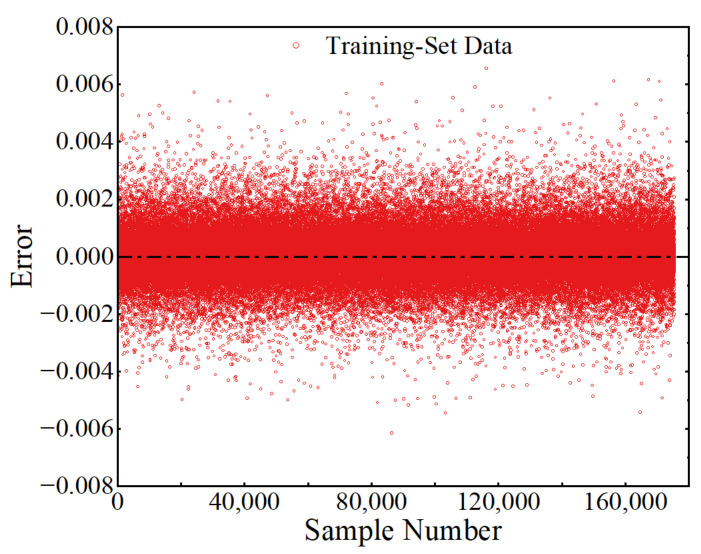
Errors of the ANN in predicting the training-set samples.

**Figure 14:**
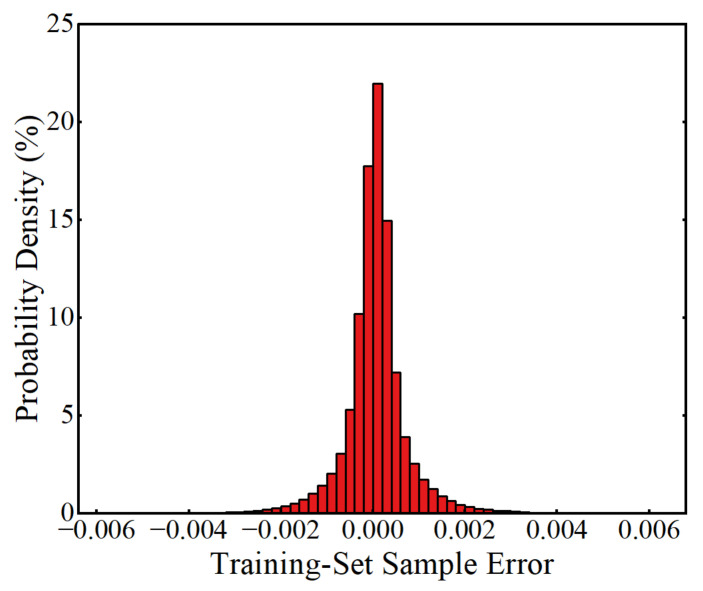
Probability density of the errors in predicting the training-set samples.

After training for 1000 epochs, a trained ANN was obtained to map the value of the input parameters and LSF. The value of the loss function at each epoch of the training process is shown in Figure 11, from which it can be seen that the value of MSE reached 4.80 × 10^−7^ at the completion of the training, indicating that the model training converged well. Figure 12 shows the true values and the ANN-predicted values of the LSFs in the training-set samples, from which it can be observed that the predicted LSFs are very close to the true values. The error of each sample and the probability density distribution of the errors are represented in Figure 13 and Figure 14, respectively, in which it can be seen that the trained ANN has a very good performance in predicting the LSF of the training-set samples.

**Figure 15:**
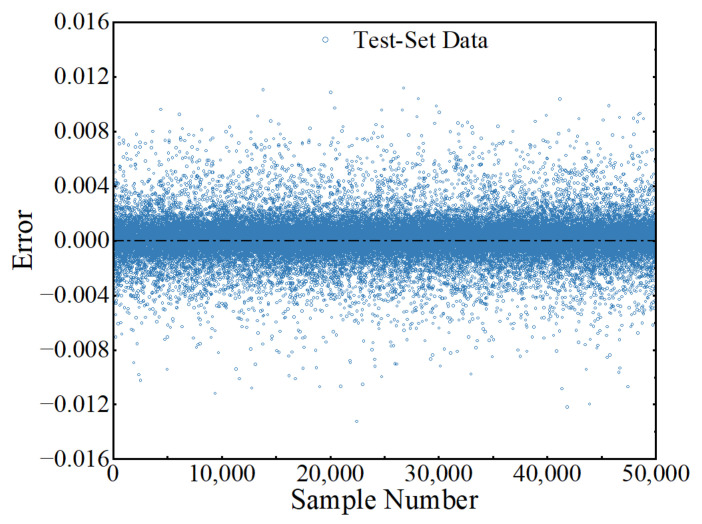
Errors in predicting the test-set samples.

**Figure 16:**
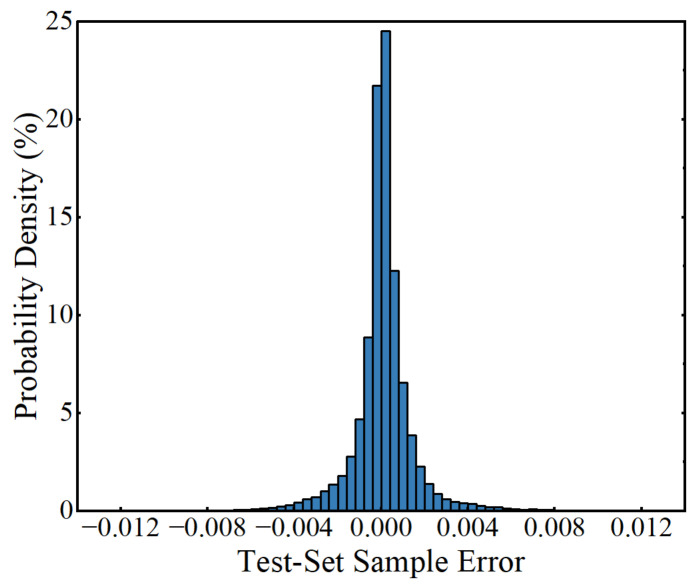
Probability density of the errors in predicting the test-set samples.

To check the robustness of the trained ANN, 50,000 (100 × 500 = 50,000) test-set samples were generated, similar to the process of generating training-set samples. It should be noted that the 100 sets of traffic-characteristic parameters of the test-set samples were randomly generated to avoid to be the same as those of the training-set sample inputs. The MSE of the ANN in predicting LSFs of the test-set samples was 1.99 × 10^−6^. The errors of the trained ANN in predicting the outputs (LSFs) of test-set samples and the corresponding probability density are represented in Figure 15 and Figure 16, respectively, from which it can be observed that the errors were between −0.0133 and 0.0112, indicating that the trained ANN has a good performance in predicting the LSF of new samples and has good robustness.

## 7. Results and Discussion

Monte Carlo Simulation (MCS) is a commonly used method for structural reliability analysis, whose reliability can be guaranteed if the amount of random samples is sufficient [28]. A sampling number of 10^6^ for MCS, which is sufficient for reliability analysis, is adopted in this study [29]. The target value of the reliability index is commonly taken as 2.0 to 3.5 for steel bridges [30], and a reliability index of 2.0 is equivalent to *P*_f_ = 2.3%. In the present research, the target probability of fatigue failure was set to 2.3% [14,15]. Through trail calculations, it was found that there was a huge gap in the computational time between the conventional Monte Carlo simulation and the ANN-MCS method for reliability analysis. For example, assuming *t* = 10 and ADTT = 3000, it takes about 8.3 h and 0.8 s to perform a reliability analysis by the conventional Monte Carlo simulation and the ANN-MCS method, respectively. Therefore, applying the ANN-MCS method to fatigue reliability analysis has a significant advantage in computational efficiency while ensuring accuracy. In the subsequent part, the effects of four key parameters, namely, the ADTT, GVWL, VR, and *a*_dtc_, on the failure probability of the considered fatigue detail are investigated.

### 7.1. Effects of the Average Number of Daily Truck Traffic (ADTT)

**Figure 17:**
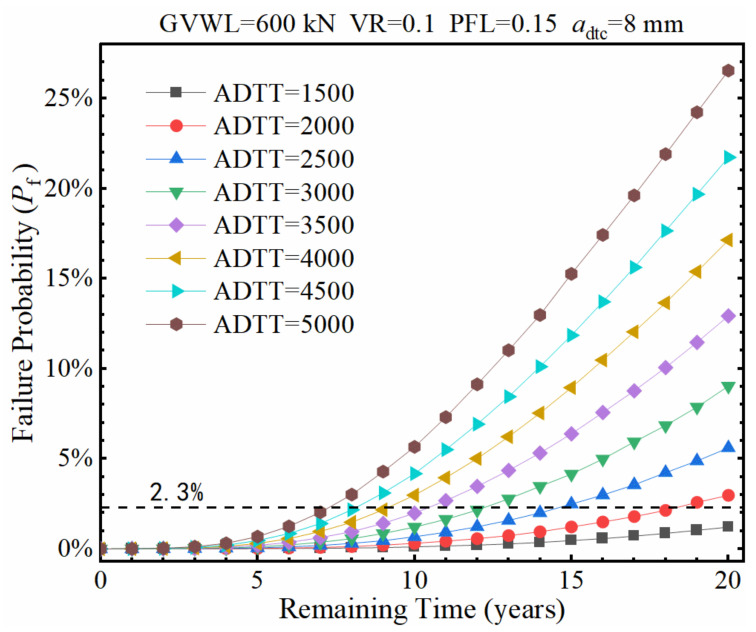
Failure probability of the fatigue detail under various ADTTs.

The failure probability (*P*_f_) of the fatigue detail under consideration was calculated under different ADTTs as the remaining service time (*t*) increases, as shown in Figure 17, in which the ADTTs ranges from 1500 to 5000 while the values of GVWL, VR, PFL, and *a*_dtc_ were adopted to be 600 kN, 0.1, 0.15, and 8 mm, respectively. It is seen from Figure 17 that the time required for the failure probability of the fatigue detail increasing to over 2.3% reduces significantly as the ADTT increases. Specifically, the required time decreases from over 20 years to around 7 years as the ADTT increases from 1500 to 5000.

### 7.2. Effects of the Gross Vehicle Weight Limit (GVWL)

**Figure 18:**
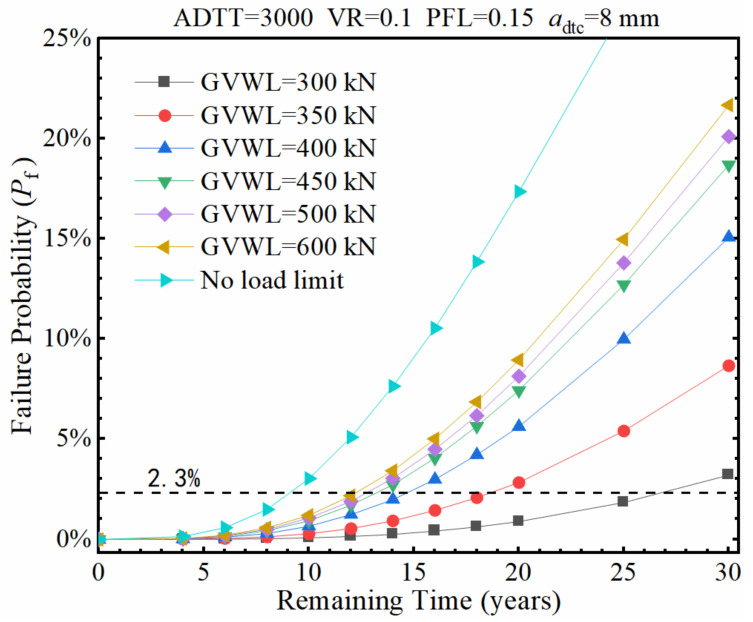
Failure probability of the fatigue detail under various GVWLs.

The failure probability (*P*_f_) of the fatigue detail under consideration was calculated under different GVWLs as the remaining service time increases, as shown in Figure 18, in which the GVWL ranges from 300 kN to 500 kN while the values of ADTT, VR, PFL, and *a*_dtc_ were adopted to be 3000, 0.1, 0.15, and 8 mm, respectively. It should be noted that no load limit was also considered and illustrated in Figure 18 for comparison. It can be observed from Figure 18 that the implementation of truck weight limits can effectively extend the remaining service time of the bridge. Specifically, even if the GVW is limited to 600 kN, the remaining service time with a 2.3% failure probability can also be effectively increased from 9 years to 12 years compared to no weight limit. Furthermore, if the GVWL is set to 350 kN, the service time can be significantly extended to 18 years. In addition, it is interesting to find that when the GVWL is set to 450, 500, and 600 kN, respectively, there is not much difference in the remaining service time, which may be due to the fact that the proportion of trucks with the GVW ranging from 450 to 600 kN is small.

### 7.3. Effects of the Violation Rate (VR)

**Figure 19:**
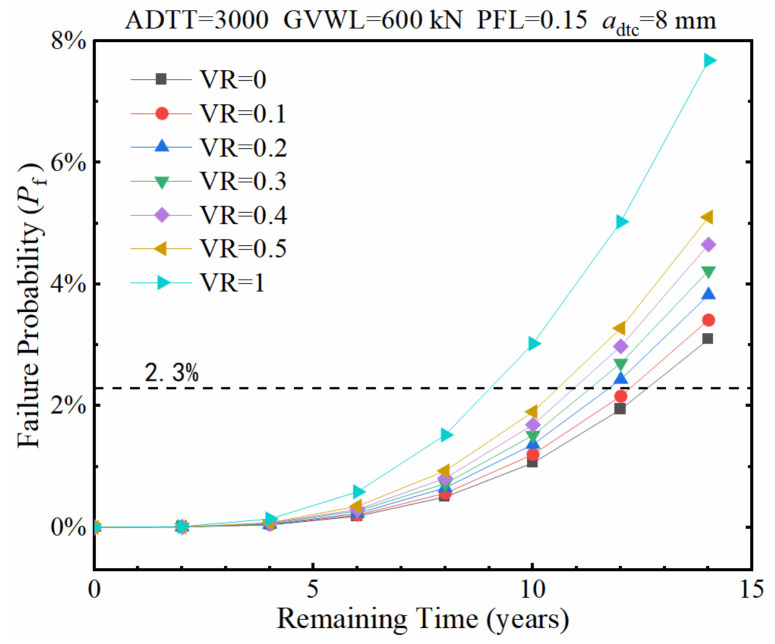
Failure probability of the fatigue detail under various VRs when the GVWL equals 600 kN.

**Figure 20:**
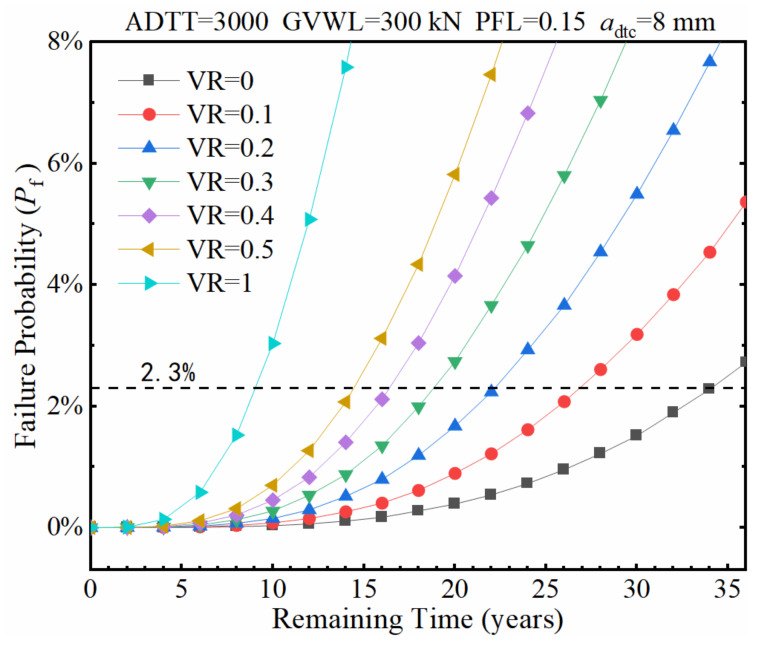
Failure probability of the fatigue detail under various VRs when the GVWL equals 300 kN.

With the GVWL preset to 600 kN and 300 kN, the effects of VR on the fatigue failure probability of the considered fatigue detail are shown in Figure 19 and Figure 20, respectively. It is found from Figure 19 and Figure 20 that the remaining service time with a failure probability of 2.3% sharply reduces from 27 years to 14.5 years when the VR increases from 0.1 to 0.5 if the GVWL is taken a value of 300 kN. However, the remaining service time with a failure probability of 2.3% only decreases from 12 years to 10.3 years when the VR increases from 0.1 to 0.5 if the GVWL is adopted to be 600 kN. This indicates that the role played by the VR is closely related to the value of GVWL, and high VR values lead to significant increases in the fatigue failure probability when the GVWL is set low.

### 7.4. Effects of the Detected Crack Size (a_dtc_)

**Figure 21:**
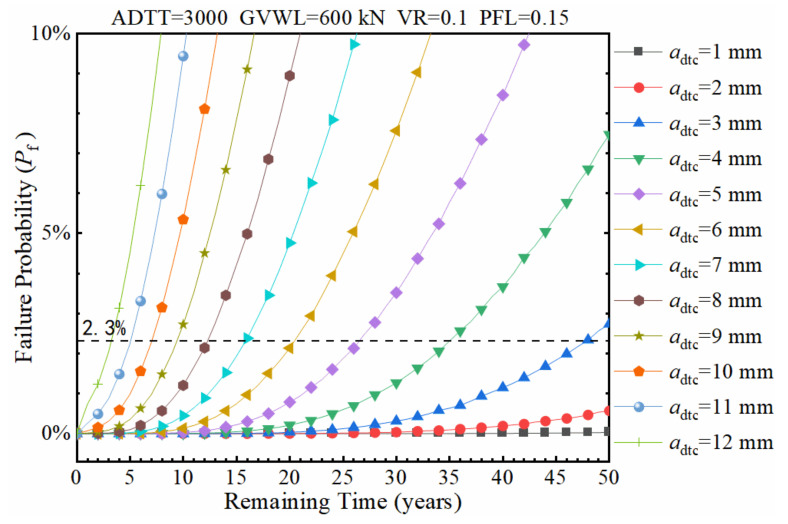
The failure probability of the fatigue detail under various detected crack sizes.

In the present research, the actual crack size was assumed to follow a lognormal distribution with *a*_dtc_ as the mean value and 0.25 as the CoV. When ADTT = 3000, GVWL = 600 kN, VR = 0.1, and PFL = 0.15, the failure probability was calculated under different *a*_dtc_ values, as exhibited in Figure 21. It is found from Figure 21 that the detected crack size *a*_dtc_ has a considerable impact on the failure probability of the bridge. If the *a*_dtc_ is less than or equal to 2 mm, the expected remaining service time with a 2.3% failure probability is greater than 50 years, which indicates that the fatigue failure is unlikely to happen in the short term. However, if the *a*_dtc_ exceeds 7 mm, the estimated remaining service time with a 2.3% failure probability is less than 16 years. Furthermore, while the *a*_dtc_ is greater than or equal to 9 mm, the predicted remaining service life with a 2.3% failure probability is less than 10 years, and corresponding measures should be taken to control the increase in the crack size, such as rehabilitation, traffic control, and truck weight limit enforcement.

### 7.5. Truck Weight Limits

**Figure 22:**
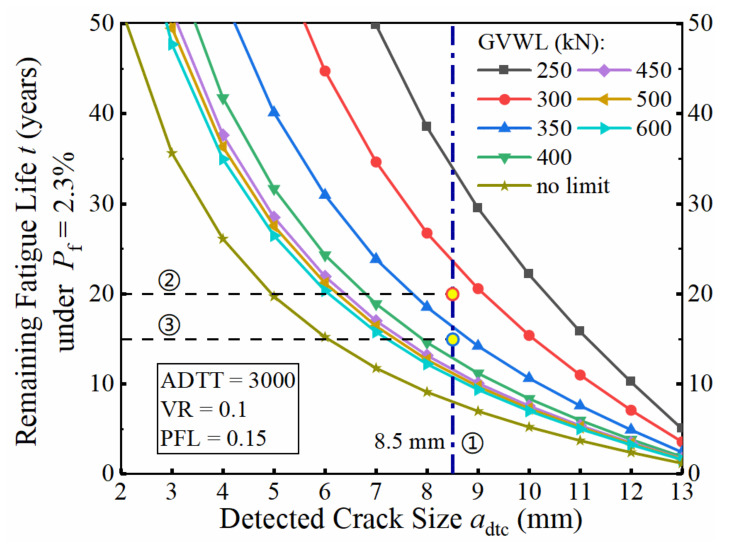
Relationship between the remaining fatigue life and the detected crack size when VR = 0.1, ADTT = 3000, PFL = 0.15, and *P*_f_ = 2.3%.

A method of setting the gross vehicle weight limit for steel bridges with cracks is proposed in this section. When the target failure probability of fatigue detail is set to 2.3%, the recommended value of the GVWL can be determined based on the four parameters, including the average daily truck traffic (ADTT), violation rate (VR), detected crack size (*a*_dtc_), and remaining service time (*t*). The relationship between the remaining fatigue life (*t*) and the parameters under consideration was obtained by the ANN-MCS method. For example, assuming VR = 0.1, ADTT = 3000, PFL = 0.15, and *P*_f_ = 2.3%, the relationship between *t* and *a*_dtc_ under different values of GVWL are presented as the solid lines in Figure 22. Based on Figure 22, a reasonable GVWL can be determined based on the detected crack size and the required remaining fatigue life. First, find the detected crack size from the abscissa, such as 8.5 mm, and make a vertical line marked as Line①, as shown by the dash-dotted line in Figure 22. Then, find the expected remaining service time (*t*) from the ordinate and draw a horizontal line, and then find the intersection point with the Line① made in the first step. Finally, find the nearest solid line right above the intersection and the GVWL value corresponding to this line is the accepted limit for gross vehicle weight.

For example, if *a*_dtc_ is 8.5 mm and the expected remaining service time (*t*) is 20 years, draw a horizontal Line②, and it can be seen that the solid line representing GVWL = 300 kN is just above the intersection of Line② and Line①. Therefore, the gross vehicle weight limit could set to 300 kN. Similarly, if the expected remaining service time is 15 years, draw the horizontal Line③ and it can be found that the solid line denoting GVWL = 350 kN is just above the intersection of Line③ and Line①. Therefore, the accepted gross vehicle weight limit, in this case, is 350 kN.

## 8. Summary and Conclusions

Very few previous studies investigated the influences of the gross vehicle weight limit (GVWL) on the fatigue failure probability of in-service steel bridges with cracks. In this study, a new framework was proposed for fatigue reliability analysis of steel bridges with cracks, in which the computational efficiency in calculating the fatigue failure probability was significantly improved through introducing the ANN. The framework proposed in the present study can help predict the fatigue failure probability of steel bridges with cracks based on four key parameters, including the average daily truck traffic, gross vehicle weight limit, violation rate, and crack size. In addition, with the goal of controlling fatigue failure probability to be less than the target value of 2.3%, a new method was proposed to set the gross vehicle weight limit for in-service steel bridges with cracks based on the detected crack size and the desired remaining service time.

A random traffic load model under weight limits was established based on the transportation survey data from Wisconsin of the United States. A typical 30.4-m-span steel-concrete composite girder bridge with a crack at the bottom flange of the girder was utilized as an example to illustrate the feasibility of the suggested framework for fatigue reliability analysis. Results show that when using the Monte Carlo method for fatigue reliability analysis of steel bridges with cracks, the conventional process of calculating the limit state function is time-consuming. As a substitution, the proposed ANN-MCS method shows great performance in terms of high accuracy, high efficiency, and good robustness in calculating the limit state function. Through parametric analysis, it is observed that both the flow-controlling measures and gross vehicle weight limits are effective for prolonging the remaining fatigue life of steel bridges with cracks. Strict implementation of the gross vehicle weight limit is essential because high violation rates can lead to significant increases in the probability of fatigue failure. In addition, the probability of fatigue failure is particularly sensitive to the detected crack size and when the size is greater than 9 mm, the predicted remaining service life with a failure probability of 2.3% is less than 10 years and measures should be taken in time to control the increase in the crack size.

It should be noted that only the vehicle-induced fatigue damage was taken into consideration in the present study, and the influences of many other factors, including corrosion and seismic loads, on the fatigue damage were ignored and ought to be given attention in future research.

## Figures and Tables

**Table 1:** Statistical parameters of the traffic load in Wisconsin.

Truck Class	*P* (%)	*μ*_1_ (kN)	*σ*_1_ (kN)	*p*_1_	*μ*_2_ (kN)	*σ*_2_ (kN)	*p*_2_
5	28.88	72.81	18.91	0.37	106.91	31.27	0.63
6	8.48	126.39	30.39	0.83	217.75	26.66	0.17
7	2.15	95.01	22.55	0.02	259.42	60.96	0.98
8	9.63	134.28	22.49	0.49	185.34	40.5	0.51
9	45.08	163.61	30.89	0.21	304.28	65.69	0.79
10	0.8	170.28	18.91	0.04	343.43	82.29	0.96
11	0.63	227.62	77.76	0.92	287.53	4.54	0.08
13	4.34	538.42	141.21	0.53	804.55	148.68	0.47

**Table 2:** Statistical distribution of parameters related to crack growth.

Parameter	Distribution Type	Mean Value	CoV
*a*_0_	Lognormal	*a*_dtc_	0.25
*C*	Lognormal	$3{.92\;\times \;10}^{-12}$	0.63
*m*	Deterministic	3	-

**Table 3:** Ranges of input parameters.

Parameter	Lower Bound	Upper Bound	Unit
ADTT	1500	5000	-
GVWL	250	1200	kN
PFL	0	0.4	-
VR	0	0.5	-
*t*	0	50	years
*a*_0_	0.6	22.2	mm
*C*	0	2 × 10^−11^	(${\mathrm{MPa}}^{3}\sqrt{m}$)^−1^
